# Insanity in Paris

**Published:** 1860-09

**Authors:** 


*Insanity in Paris.*—It is stated by the French press that lunacy is much on the increase in Paris. It is certain that recently a considerable number of eccentric and insane persons have publicly exhibited their peculiararities in such a manner as to call for restraint. This may be an accidental and temporary condition of affairs. Twice during the last few days, the police have arrested three persons who were openly committing acts of insanity in public places. On Saturday week, three lunatics successively applied for admission at the Tuileries, seeking an audience of the Emperor Napoleon on various pretences.—*Lancet.*

